# Machine learning for the prediction of acute kidney injury in patients after cardiac surgery

**DOI:** 10.3389/fsurg.2022.946610

**Published:** 2022-09-07

**Authors:** Xin Xue, Zhiyong Liu, Tao Xue, Wen Chen, Xin Chen

**Affiliations:** ^1^ Department of Cardiothoracic Surgery, Zhongda Hospital, School of Medicine, Southeast University, Nanjing, China; ^2^ Department of Cardiothoracic Surgery, School of Medicine, Southeast University, Nanjing, China; ^3^ Department of Thoracic and Cardiovascular Surgery, Nanjing First Hospital, Nanjing Medical University, Nanjing, China

**Keywords:** cardiac surgery, acute kidney injury, machine learning, random forest, risk model

## Abstract

Cardiac surgery-associated acute kidney injury (CSA-AKI) is the most prevalent major complication of cardiac surgery and exerts a negative effect on a patient's prognosis, thereby leading to mortality. Although several risk assessment models have been developed for patients undergoing cardiac surgery, their performances are unsatisfactory. In this study, a machine learning algorithm was employed to obtain better predictive power for CSA-AKI outcomes relative to statistical analysis. In addition, random forest (RF), logistic regression with LASSO regularization, extreme gradient boosting (Xgboost), and support vector machine (SVM) methods were employed for feature selection and model training. Moreover, the calibration capacity and differentiation ability of the model was assessed using net reclassification improvement (NRI) along with Brier scores and receiver operating characteristic (ROC) curves, respectively. A total of 44 patients suffered AKI after surgery. Fatty acid-binding protein (FABP), hemojuvelin (HJV), neutrophil gelatinase-associated lipocalin (NGAL), mechanical ventilation time, and troponin I (TnI) were correlated significantly with the incidence of AKI. RF was the best model for predicting AKI (Brier score: 0.137, NRI: 0.221), evidenced by an AUC value of 0.858 [95% confidence interval (CI): 0.792–0.923]. Overall, RF exhibited the best performance as compared to other machine learning algorithms. These results thus provide new insights into the early identification of CSA-AKI.

## Introduction

Acute kidney injury (AKI) is the most frequent major complication of cardiac surgery (1). Annually, over two million cardiac procedures are performed globally, and the incidence of CSA-AKI ranges from 5% to 42.3% (2, 3). Cardiac surgery is the second most frequent cause underlying AKI in the intensive care unit (ICU), resulting in three- to eight-fold higher perioperative mortality, prolonged ICU and hospital stay, and increased healthcare costs for patients with severe AKI (4, 5). At present, renal replacement therapy is the only option available for patients with advanced severe CSA-AKI due to the lack of effective therapies. Therefore, early detection of AKI will provide clinicians with the necessary guidance for its prevention and management (6). Existing clinical risk assessments are based on changes in the levels of serum creatinine (Scr) and reported risk factors. However, these cannot accurately identify patients with AKI due to limitations in their sensitivity and specificity, thus resulting in missed optimal treatment timing. Accumulating evidence focuses on novel biomarkers and clinical prediction models to identify risk factors for AKI and improve its diagnostic efficiency and accuracy (7–10).

Predictive models for cardiovascular surgery are critical for patient selection, risk stratification, tailoring treatment, and prognostic prediction. Accurate perioperative risk prediction for complications such as AKI may help in better informing these patients and their families of the risks and assist in clinical management (11). Many perioperative prediction models for AKI have been developed in recent years. However, the general utility of these models is poor due to differences in variable selection (7, 12, 13). Consequently, there is neither a consensus nor guidelines recommending the use of the existing predictive models for AKI after cardiac surgery.

Machine learning algorithms and advanced statistical tools can be used to predict the outcome of complex datasets based on iterative learning, thereby making these models more accurate and stable through the selection of variable features (14, 15). Therefore, machine learning algorithms were used in this study to develop and validate feature variables for predicting perioperative AKI risk and generating robust prediction models, in addition to traditional logistic regression analyses. We collected the patients' perioperative demographic characteristics, clinical laboratory data, and intraoperative and postoperative clinical data. Subsequently, a feature selection machine learning strategy was used to develop multivariate models to predict the risk of severe CSA-AKI.

## Material and methods

### Data sources

Patients who were admitted to the ICU (aged 18 years or above) after cardiac surgery by cardiopulmonary bypass (CPB) at the Department of Cardiothoracic Surgery of Nanjing First Hospital between 1 December 2019, and 30 April 2020, were enrolled. The cardiac surgery procedures included valve replacement, coronary artery bypass surgery, large vessel surgery, combined surgery, and congenital heart disease correction surgery.

The exclusion criteria in this study were as follows: (1) renal insufficiency or acute and chronic kidney disease; (2) recent administration of renal impairment drugs or glucocorticoid drugs; (3) recent or postoperative concomitant urinary tract infection; (4) preoperative hemodynamic instability, and (5) emergency surgery.

All patients provided written informed consent before participating in this study. The study design was approved by the Medical Ethics Committee of Nanjing First Hospital (KY20190404-03-KS-01) and adhered to the *Declaration of Helsinki*.

### Diagnostic criteria and outcome definitions

The outcome of interest was the occurrence of AKI during the perioperative period. AKI was defined as follows: the diagnosis was confirmed according to the latest diagnostic criteria for AKI in the 2012 KDIGO guidelines (16) if one of the following conditions was met: (1) Scr elevation ≥0.3 mg/dl ≥26.5 µmol/L within 48 h; (2) known or presumed Scr elevation ≥1.5 times baseline occurring within seven days, or (3) sustained six h urine volume <0.5 ml/kg/h. Baseline preoperative Scr value was defined as the last Scr value detected within seven days before performing cardiac surgery with CPB.

### Model development

First, support vector machine (SVM), least absolute shrinkage and selection operator (LASSO) regression, extreme gradient boosting (XGBoost), and Random Forest and Boruta (RFB) algorithms were used for filtering out the crucial clinical variables. Subsequently, the feature variables were derived to construct and validate the model.

A stratified five-fold cross-validation was performed to obtain the derivation and validation cohorts. The study population was randomly classified into five subsets with similar event rates. Four subsets (80%) were combined to form the derivation cohort, whereas the remaining (20%) was retained as the validation set. This process was repeated five times for each outcome so that each subset could serve as the validation set, thus accounting for inter-patient variability and providing a risk estimate for all cases. Since five numerical features (fatty acid-binding protein [FABP], NT-prBNP, troponin I (TnI), ultrafiltration volume, and urine dropout) had <20% missing data, predictive mean matching was used to fill in the incomplete information.

Four common machine learning algorithms and conventional logistic regression were employed to train the models, including logistic regression with LASSO regularization (logistic LASSO), logistic regression with forwarding selection variables, RF, SVM, and xgboost. The basic GLM functions were used for logistic regression. In addition, packages including glmnet, randomForest, xgboost, and e1071 were used for LASSO (17), RF (18), Xgboost (15), and SVM (19) analyses, respectively. Logistic regression was performed along with forward stepwise selection to determine the average C-index improvement for each added variable. In high-dimensional problems, backward selection techniques may be prone to greater noise. In contrast, forward selection leads to strong theoretical guarantees and excellent empirical behavior. In addition, logistic LASSO models were constructed by five-rule cross-validation and default lambda based on minimum classification errors. The number of trees for RF was 1,000, with 50 perturbation counts. Moreover, the number of trees for xgboost was 100, with a learning rate of 1 per tree, a maximum depth of 2 and trained through 50 iterations. Finally, two different kernels (linear and radial-based functions) were used in the SVM algorithm to obtain the separation function.

### Model performance

Receiver operating characteristic (ROC) curves were used to estimate the model's discrimination capacity by calculating its area under the curve (AUC). Confusion matrices were plotted to assess the model's effectiveness based on multiple metrics. In addition, net reclassification improvement (NRI) was used to assess the correct reassignment between risk categories (20). Predictive calibration plots were used to plot the average risk scores relative to the observed outcome rates. In addition, the probability of the optimal performance model was assessed using the Brier score, which was defined as the mean squared error between the observed and predicted outcomes. The Brier score ranges from 0 to 1.00, with the former representing the best possible calibration. The prediction for each patient was plotted in the order of their risk to assess the predictive distribution of the model (15). Furthermore, decision curve analysis (DCA) curves were plotted to assess the discriminability of each selected factor to predict severe CSA-AKI (21). Next, the nomogram of the optimal model was constructed using the “rms” package in R. Finally, the Hosmer–Lemeshow test was conducted to assess the fit of the nomogram (22).

### Statistical analyses

All graphs were plotted and analyses were performed using the R software (version: 4.1.0). Continuous variables were compared by two-tailed *t*-tests, whereas Fisher's exact test was used for categorical data. The significance level was set at *P* < 0.05 unless specified otherwise.

## Results

### Patient characteristics and outcomes

A total of 215 patients underwent cardiac surgery under CPB between December 1, 2019, and April 30, 2020, of which 135 were enrolled (the flow chart in Supplementary Figure S1 details inclusion and exclusion criteria and selection of the final study cohort for further analyses). According to the KDIGO clinical practice guidelines, 44 patients suffered from hospital-acquired AKI within one week of cardiac surgery; the incidence rate was 32.59%. Baseline characteristics of the patients are shown in Table 1. Moreover, the non-AKI and AKI groups included 49.5% and 65.9% of men and the median EF was 60.97% and 58.14%, respectively. The mean ICU length of stay and mean mechanical ventilation time were longer in the AKI group than those in the non-AKI group (3.05 vs. 1.48, *P* < 0.001; 21.27 vs. 9.63, *P* < 0.001). However, no significant differences were observed in Cleveland clinical scores between the two groups.

**Table 1:** Demographic and clinical characteristics of patients with or without AKI after cardiac surgery.

Variable	non-AKI (N = 91)	AKI (N = 44)	P Value
Gender (%)			0.106
Male	45 (49.5)	29 (65.9)	
Female	46 (50.5)	15 (34.1)	
Age (mean (SD))	59.58 (11.33)	62.52 (10.35)	0.149
Height (mean (SD))	163.51 (8.77)	164.39 (8.79)	0.585
Weight (mean (SD))	65.03 (10.76)	65.93 (10.13)	0.644
BMI (mean (SD))	1.79 (0.17)	1.81 (0.16)	0.593
Smoke (%)			0.148
No	72 (79.1)	29 (65.9)	
Yes	19 (20.9)	15 (34.1)	
Drink (%)			0.141
No	88 (96.7)	39 (88.6)	
Yes	3 (3.3)	5 (11.4)	
Diabetes (%)			1.000
No	69 (75.8)	33 (75.0)	
Yes	22 (24.2)	11 (25.0)	
Hypertension (%)			0.634
No	40 (44.0)	22 (50.0)	
Yes	51 (56.0)	22 (50.0)	
CRF (%)			0.141
No	88 (96.7)	39 (88.6)	
Yes	3 (3.3)	5 (11.4)	
AF (%)			1.000
No	75 (82.4)	37 (84.1)	
Yes	16 (17.6)	7 (15.9)	
Hb (mean (SD))	131.65 (18.38)	134.36 (21.74)	0.453
Wb (mean (SD))	39.99 (3.01)	38.88 (3.26)	0.052
hCT (mean (SD))	25.38 (3.65)	24.61 (4.90)	0.307
CVP (mean (SD))	8.32 (3.18)	9.02 (3.94)	0.268
EF (mean (SD))	60.97 (7.89)	58.14 (9.18)	0.067
TnI (mean (SD))	0.70 (1.62)	2.06 (2.85)	0.001
FABP (mean (SD))	3.71 (2.35)	6.28 (3.53)	<0.001
NT-prBNP (mean (SD))	556.15 (1092.79)	947.94 (735.79)	0.033
NGAL (mean (SD))	64.53 (18.77)	85.04 (20.96)	<0.001
HJV (mean (SD))	53.72 (15.52)	65.31 (18.12)	<0.001
DKK3 (mean (SD))	1073.72 (364.45)	1265.71 (400.68)	0.006
CAG (%)			0.509
No	13 (14.3)	9 (20.5)	
Yes	78 (85.7)	35 (79.5)	
Interval Time (mean (SD))	6.15 (6.24)	7.93 (15.98)	0.355
CPBT (mean (SD))	108.96 (33.15)	123.41 (43.37)	0.034
Urine dropout (mean (SD))	254.51 (198.94)	213.98 (173.72)	0.25
Ultrafiltration volume (mean (SD))	1586.37 (1014.57)	1967.05 (1175.57)	0.055
Aortic occlusion time (mean (SD))	74.13 (25.55)	80.93 (28.84)	0.167
Erythrocyte infusion (mean (SD))	0.10 (0.30)	0.18 (0.39)	0.176
Hospitalization time (mean (SD))	17.38 (7.19)	18.66 (5.85)	0.308
ICU length of stay (mean (SD))	1.48 (1.06)	3.05 (3.65)	<0.001
Mechanical ventilation time (mean (SD))	9.63 (4.87)	21.27 (30.23)	<0.001
Cleveland (mean (SD))	1.68 (1.39)	2.02 (1.07)	0.153

Note: Single operation is either coronary artery bypass grafting, heart valve surgery, great vascular surgery, valve replacement or valve plasty.

### Machine learning algorithms for variable selection

**Figure 1:**
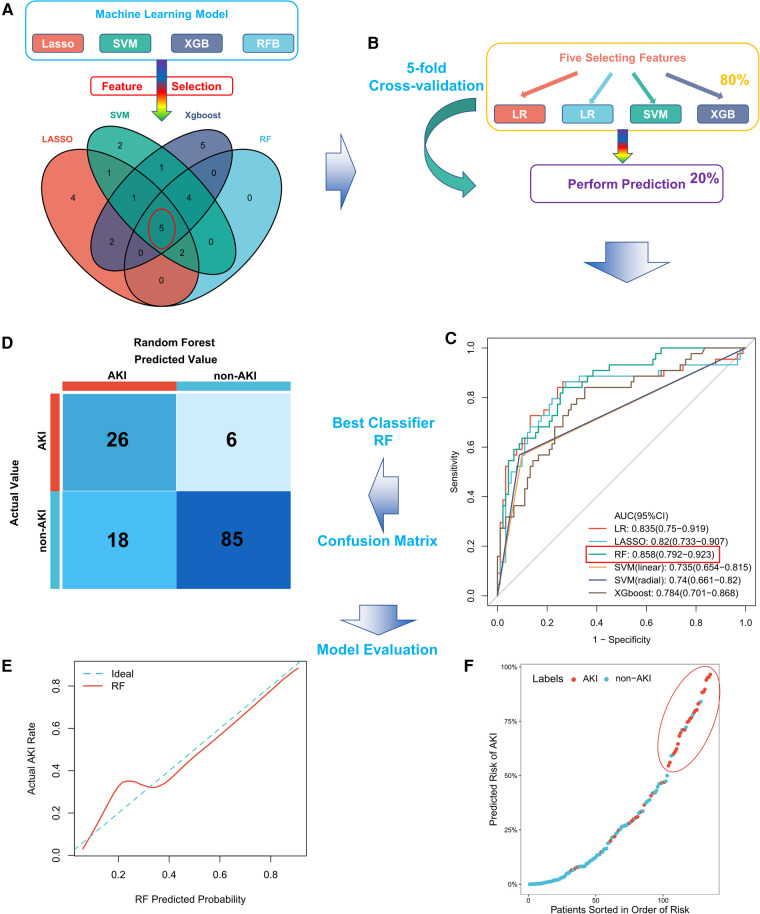
Construction and evaluation of the CSA-AKI prediction model. (**A**) The five most important clinical features screened using four machine learning algorithms in the entire cohort; (**B**) schematic diagram of six machine learning algorithms in the training set trained and validated for stable clinical models by five-fold cross-validation; (**C**) comparison of AUC values among machine learning models, with RF having the largest AUC value; (**D**) confusion matrix of the best model (RF) in the entire cohort; (**E**) calibration curve for the RF model; (**F**) distribution of predicted patient risk of CSA-AKI. CSA-AKI: cardiac surgery-associated acute kidney injury.

Thirty-four baseline clinical characteristics with at least 70% complete data were considered predictors of CSA-AKI. Table 2 summarizes the simple logistic regression and ROC analyses of clinical characteristics. Four machine learning algorithms (LASSO, RFB, SVM, and XGBoost) were used for the entire dataset to identify the most important clinical variables for AKI prediction, yielding 15, 11, 16, and 18 clinical variables, respectively (Supplementary Figure S2, detailed list is provided in Table S1). In addition, five commonly shared clinical features among the four algorithms were included as variables in the final model, comprising FABP, hemojuvelin (HJV), mechanical ventilation time, neutrophil gelatinase-associated lipocalin (NGAL), and TnI (Figure 1A); their risk ratios were statistically significant (*P* < 0.05).

**Table 2:** Univariate, multivariate logistic regression and ROC analysis.

Variable	Univariate analysis	NA	Multivariate analysis	NA	Roc analysis
	OR (95% CI)	*P*-value	OR (95% CI)	*P*-value	AUC (95% CI)
Gender	0.506 (0.240–1.068)	0.074	NA		0.582 (0.495–0.67)
Age	1.026 (0.991–1.062)	0.150	NA		0.573 (0.467–0.678)
Height	1.012 (0.971–1.055)	0.582	NA		0.537 (0.433–0.64)
Weight	1.008 (0.974–1.043)	0.641	NA		0.536 (0.434–0.639)
BMI	1.824 (0.208–16.020)	0.587	NA		0.542 (0.439–0.645)
Smoke	1.960 (0.878–4.373)	0.100	NA		0.566 (0.484–0.648)
Drink	3.761 (0.856–16.523)	0.079	NA		0.54 (0.489–0.591)
Diabetes	1.045 (0.454–2.408)	0.917	NA		0.504 (0.426–0.583)
Hypertension	0.784 (0.381–1.614)	0.509	NA		0.53 (0.44–0.621)
CRF	3.761 (0.856–16.523)	0.079	NA		0.54 (0.489–0.591)
AF	0.887 (0.336–2.343)	0.809	NA		0.492 (0.424–0.559)
Hb	1.007 (0.989–1.026)	0.448	NA		0.525 (0.415–0.635)
Wb	0.891 (0.791–1.003)	0.056	NA		0.623 (0.521–0.725)
hCT	0.953 (0.869–1.045)	0.306	NA		0.574 (0.468–0.681)
CVP	1.062 (0.955–1.182)	0.267	NA		0.544 (0.434–0.655)
EF	0.962 (0.922–1.003)	0.072	NA		0.611 (0.51–0.712)
TnI	1.415 (1.106–1.810)	0.006	1.619 (1.187–2.208)	0.002	0.718 (0.626–0.811)
FABP	1.336 (1.164–1.533)	<0.001	1.105 (0.925–1.320)	0.273	0.771 (0.688–0.854)
NT-prBNP	1.001 (1.000–1.001)	0.077	NA		0.73 (0.634–0.826)
NGAL	1.057 (1.033–1.083)	<0.001	1.051 (1.021–1.082)	0.001	0.772 (0.684–0.86)
HJV	1.048 (1.021–1.076)	<0.001	1.034 (0.999–1.071)	0.060	0.714 (0.616–0.812)
DKK3	1.001 (1.000–1.003)	0.008	1.001 (0.999–1.002)	0.267	0.669 (0.571–0.767)
CAG	0.648 (0.253–1.657)	0.365	NA		0.469 (0.399–0.539)
Interval time	1.015 (0.982–1.050)	0.370	NA		0.471 (0.367–0.574)
CPBT	1.010 (1.001–1.020)	0.037	1.012 (0.998–1.026)	0.082	0.588 (0.481–0.696)
Urine dropout	0.999 (0.997–1.001)	0.253	NA		0.581 (0.478–0.685)
Ultrafiltration volume	1.000 (1.000–1.001)	0.058	NA		0.597 (0.494–0.701)
Aortic occlusion time	1.010 (0.996–1.023)	0.168	NA		0.558 (0.452–0.663)
Erythrocyte infusion	2.025 (0.723–5.670)	0.179	NA		0.541 (0.476–0.607)
Hospitalization time	1.027 (0.975–1.082)	0.314	NA		0.594 (0.492–0.696)
ICU length of stay	1.624 (1.178–2.239)	0.003	0.989 (0.638–1.535)	0.962	0.703 (0.613–0.793)
Mechanical ventilation time	1.084 (1.015–1.157)	0.016	1.087 (0.983–1.203)	0.104	0.661 (0.554–0.769)
Cleveland	1.223 (0.926–1.615)	0.156	NA		0.609 (0.513–0.705)

### Machine learning algorithms for outcome prediction

We constructed a machine learning classifier by five-rule cross-validation (Figure 1B). Subsequently, their performances were evaluated using ROC curves (Figure 1C). The classifier trained based on five clinical features could discriminate patients with CSA-AKI accurately. RF exhibited the best performance, with an AUC value of 0.858 (95% CI, 0.792–0.923). The specific evaluation results of the six algorithms are shown in Table 3. Among them, RF showed the best overall performance with an accuracy of 0.822 for predicting CSA-AKI. Since CSA-AKI is a clinical emergency, there is a need to accurately identify patients who are likely to develop CSA-AKI. Thus, recall is also a crucial indicator. The RF model also had the highest recall value (0.591). Furthermore, the RF model had the highest Kolmogorov–Smirnov (KS) value (0.600) relative to other models, suggesting a leading advantage in differentiating patients with CSA-AKI. RF also exhibited the greatest improvement in discrimination or classification of CSA-AKI as compared to conventional logistic regression (NRI = 0.221). The confusion matrix of the optimal model is shown in Figure 1D. Detailed results of the confusion matrix for all six models are provided in Supplementary Tables S1, S2.

**Table 3:** Evaluation results of models for AKI risk of patients after cardiac surgery.

Model	Precision	Recall	F1 score	Accuracy	KS	Error	NRI
Logistic regression with a forward selection	0.765	0.591	0.667	0.807	0.577	0.193	0.000
Logistic regression with a lasso regularization	0.826	0.432	0.567	0.785	0.589	0.215	0.012
Random forest	0.812	0.591	0.684	0.822	0.600	0.178	0.221
Support vector machine (linear kernel)	0.735	0.568	0.641	0.793	0.469	0.207	0.001
Support vector machine (radial basis function)	0.758	0.568	0.649	0.800	0.471	0.200	0.002
Extreme Gradient Boosting	0.625	0.568	0.595	0.748	0.489	0.252	−0.113

Note: KS: Kolmogorov-Smirnov; NRI: net reclassification improvement.

### Calibration of models and predictive distributions

The final RF model was well calibrated. The mean Brier score of the model for predicting CSA-AKI was 0.137 (close to 0), indicating a well-calibrated model. Figure 1E shows the calibration curve for the model. The prediction distribution plot of the RF model incorporating patients sorted by risk order suggested positive clustering of patients with CSA-AKI (Figure 1F). Therefore, the RF model could accurately stratify the patients at risk of developing CSA-AKI.

### Construction of tools for patient classification

**Figure 2:**
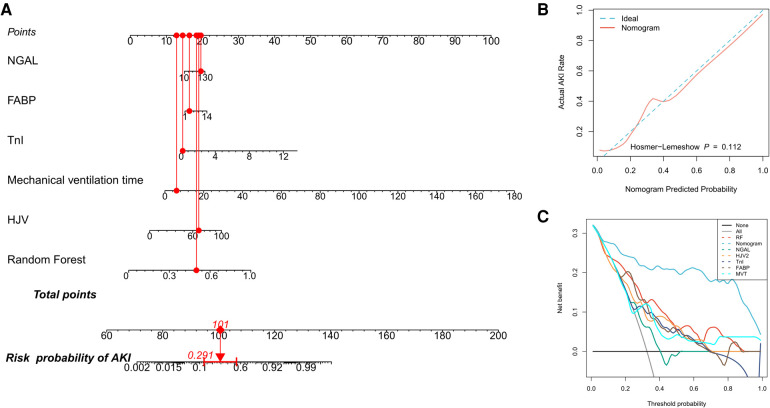
Quantifying patients’ risk of CSA-AKI. (**A**) Nomogram used to quantify the risk of CSA-AKI in patients. Redline presents the detailed score of a certain patient, with a total of 101 points and a 29.1% risk of developing CSA-AKI; (**B**) calibration curve for the nomogram; (**C**) DCA curves for performance comparisons between clinical characteristics alone, the RF model, and the nomogram by plotting the net benefit of the prediction model and clinical predictors against the threshold probabilities, wherein the horizontal axis represents the threshold (the reference probability of the patient receiving treatment) and the vertical axis represents the net benefit rate after subtracting the disadvantage from the advantage. Using the model, under the same threshold probability, a larger net benefit indicates that the patient can obtain the greatest benefit. DCA: decision curve analysis.

The nomogram is a graphical representation of the association between clinical variables and the probability of a clinical event (e.g., critical illness). In addition, it provides an intuitive way to interpret predictive models. In this study, we constructed an intuitive nomogram to specifically quantify the risk of developing CSA-AKI based on the predicted values of the RF model and their characteristic clinical variables (Figure 2A). The results of the H–L test suggested that the nomogram was well calibrated (*P* > 0.05, Figure 2B). Furthermore, DCA suggested an increased net benefit of the nomogram in predicting CSA-AKI as compared to the RF model or the characteristic clinical variables alone (Figure 2C).

## Discussion

Accurate prediction of prognosis is essential for patient-centric care, both for informing and selecting treatment strategies for inclusive decision-making. In this study, we used five alternative machine learning algorithms to characterize the risk of CSA-AKI incidence using postoperative hospitalization data in patients who underwent cardiac surgery. The RF model optimally stratified patients' risk with excellent calibration and good internal validation. In addition, clinical characteristics of patient risk that may be underestimated in clinical practice were identified, including FABP, HJV, mechanical ventilation time, NGAL, and TnI. These models provide a foundation for future clinical utility for patient care and accurate outcome risk stratification.

In addition to several published reports on predictive models or clinical scales to assess patients' risk of developing CSA-AKI, our findings extend this knowledge in several important ways. The commonly used validated scale in the clinical settings to predict CSA-AKI is the Cleveland score proposed by Thakar et al. in 2005 (23–25). However, in clinical practice, the influence of plasma and urine markers needs to be considered, in addition to the Cleveland score (26–28). Moreover, intraoperative and postoperative information is crucial to assess the risk of AKI (29). The candidate variables included in the model presented herein integrated intra- and postoperative clinical information of patients and biomarkers in urine and plasma and were based on a comprehensive set of variables that could explain the complex interactions and more accurately predict the incidence of CSA-AKI. Our final model demonstrated higher predictive efficacy as compared to traditional Cleveland scores and predictors alone. In previous studies, logistic regression models have been the traditional statistical approach for event prediction; however, machine learning can handle nonlinear interactions and combine more variables to improve predictive efficacy for dealing with data of higher dimensions (15, 30). We used advanced machine learning algorithms to assess the risk of such complex syndromes, which exhibited better performance than traditional logistic regression analysis.

Our model incorporated five common clinical characteristics and laboratory results that are available in most hospitals. However, <20% values of FABP and TnI were missing. Previous studies have not explicitly addressed the impact of missing values on predictive performances (15, 31, 32). Thus, we included missing variables systematically in the modeling approach. The final model exhibited satisfactory performance. However, it is recommended that all clinical characteristics be collected at the time of admission to gain more benefits from this model. Missing data for certain variables are inevitable in the real world, especially from small or poorly equipped hospitals. Therefore, our model also allows for interpolation and estimation of missing values.

The existing treatment modalities for CSA-AKI are severely inadequate, thus renal replacement therapy is the only available choice (6). Therefore, early intervention for patients at risk of CSA-AKI should be given high priority. In this study, we developed a model with a higher recall value to identify those at risk of CSA-AKI as early as possible. A total of 26 patients with AKI were identified in the entire cohort, of which only six were not distinguished as having AKI. The model offers more possibilities for early intervention and its clinical and economic value for postoperative cardiac management is high, especially considering the unusually rapid disease progression and the high mortality associated with CSA-AKI.

## Research limitations

This study has some limitations. First, the model did not involve subsequent baseline characteristics of the patients due to the absence of follow-up data. Although a dynamic model incorporating baseline data during hospitalization may have a better phenotype, the present model could still be used to predict the incidence of CSA-AKI with reasonable accuracy using the postoperative clinical data. Second, patient data were obtained from a single hospital. Third, the predictive accuracy using clinical medications (e.g., commonly used vasopressors, inotropic agents, and some specific drugs used during surgery) and other data (e.g., novel biomarkers, imaging, environmental factors, and atherosclerotic burden) may further improve the model. The addition of these variables could be performed in the future to improve the RF model constructed in this study. Finally, subgroup analyses based on various procedures were not performed because of the small number of patients within each subgroup. Consequently, the clinical utility of predicting postoperative CSA-AKI based on the specific procedure type is limited.

## Conclusion

In conclusion, we developed a model that integrated advanced machine learning algorithms and easily accessible patient characteristics to predict the risk of developing CSA-AKI among patients undergoing cardiac surgery. The model may provide powerful assistance to clinicians to identify patients with a higher risk of AKI early in the postoperative period. Overall, the findings of this study can assist in developing timely diagnostic and treatment strategies for the clinical management of patients undergoing cardiac surgery.

## Data Availability

The original contributions presented in the study are included in the article/**Supplementary Material**, further inquiries can be directed to the corresponding author/s.
