# Supplementary figures and images for: Machine learning for the prediction of acute kidney injury in patients after cardiac surgery
Source: Front Surg. 2022 Sep 7;9:946610. doi: 10.3389/fsurg.2022.946610 (PMC9490319; doi:10.3389/fsurg.2022.946610)

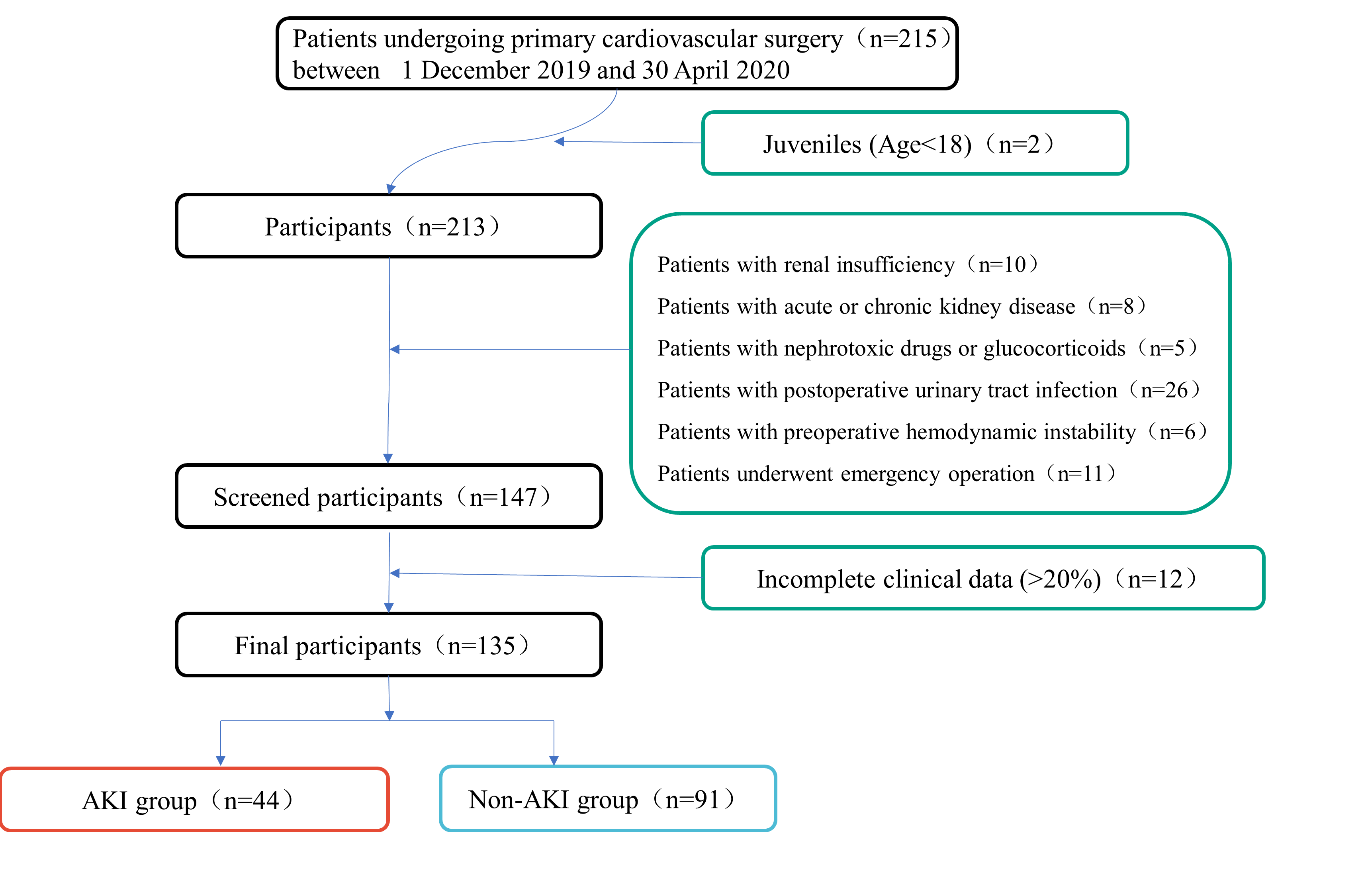

Supplement: Supplementary file 1 [file Figure_3_v1.tif]

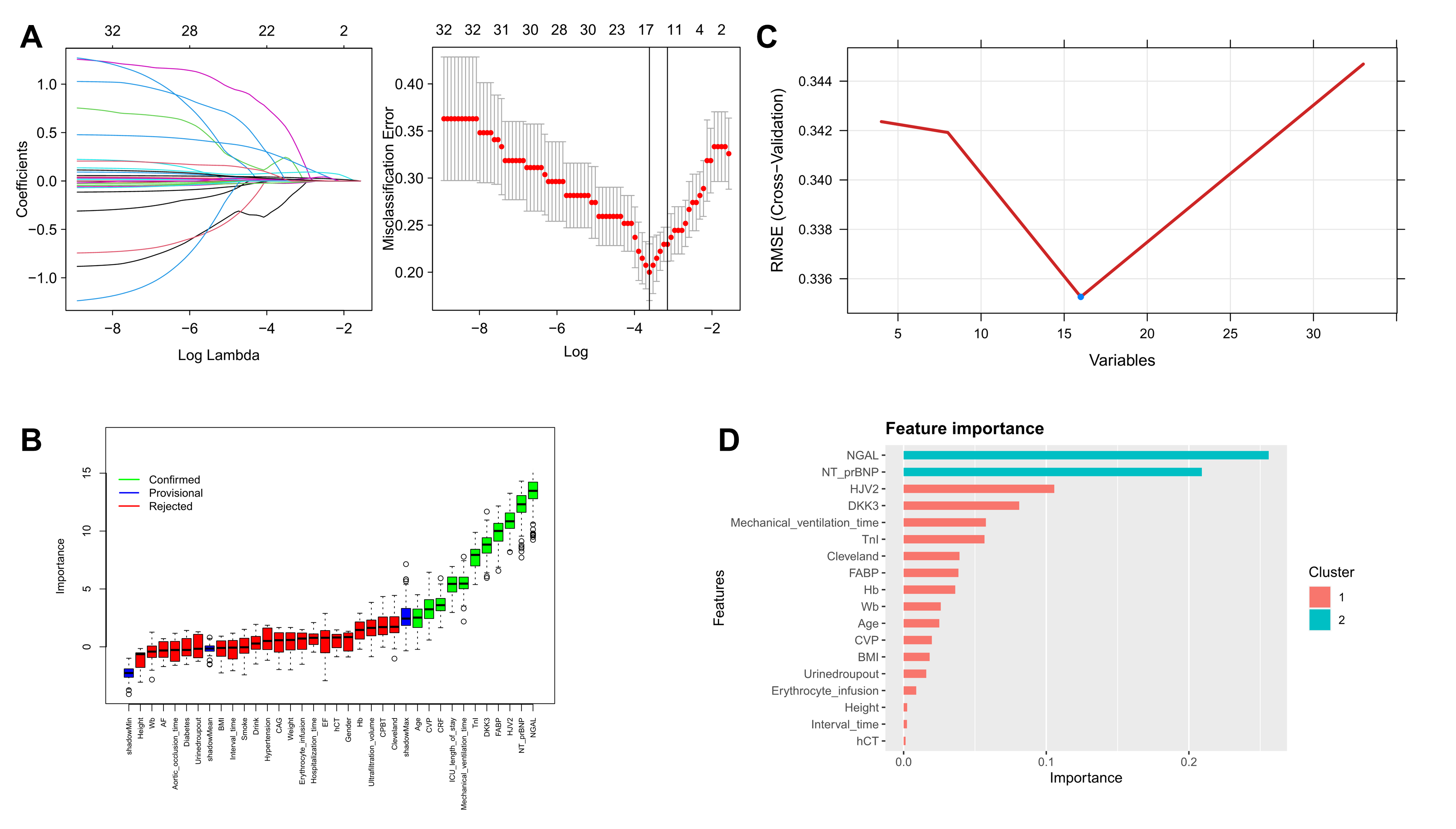

Supplement: Supplementary file 2 [file Figure_4_v1.tif]
