# Supplementary material for: Machine learning for the prediction of acute kidney injury in patients after cardiac surgery
Source: Front Surg. 2022 Sep 7;9:946610. doi: 10.3389/fsurg.2022.946610 (PMC9490319; doi:10.3389/fsurg.2022.946610)
Supplement: Supplementary file 3 [file Table_4.docx]

Table S1. The selected features of four machine learning algorithm

| **LASSO features** | **SVM featuers** | **Randomforest-**  **Boruta featuers** | **Xgboost**  **featuers** |
| --- | --- | --- | --- |
| Gender | NGAL | Age | NGAL |
| Weight | NT-prBNP | CRF | NT-prBNP |
| Drink | HJV2 | CVP | HJV2 |
| CRF | FABP | TnI | DKK3 |
| Wb | DKK3 | FABP | Mechanical ventilation time |
| EF | TnI | NT-prBNP | TnI |
| TnI | Mechanical ventilation time | NGAL | Cleveland |
| FABP | CRF | HJV2 | FABP |
| NGAL | CVP | DKK3 | Hb |
| HJV2 | Age | ICU length of stay | Wb |
| CPBT | ICU length of stay | Mechanical  ventilation time | Age |
| Erythrocyte  infusion | Hb |  | CVP |
| ICU length of stay | CPBT |  | BMI |
| Mechanical ventilation time | Cleveland |  | Urinedroupout |
| Cleveland | Ultrafiltration volume |  | Erythrocyte  infusion |
|  | Hospitalization time |  | Height |
|  |  |  | Interval time |
|  |  |  | hCT |
