# Supplementary material for: Machine learning for the prediction of acute kidney injury in patients after cardiac surgery
Source: Front Surg. 2022 Sep 7;9:946610. doi: 10.3389/fsurg.2022.946610 (PMC9490319; doi:10.3389/fsurg.2022.946610)
Supplement: Supplementary file 4 [file Table_5.docx]

Table S2. Confusion Matrix of Models for AKI Risk of Patients after Cardiac Surgery

| **model** | **TP** | **TN** | **FP** | **FN** |
| --- | --- | --- | --- | --- |
| Logistic regression with a forward selection | 26 | 83 | 8 | 18 |
| Logistic regression with a lasso regularization | 19 | 87 | 4 | 25 |
| Random forest | 26 | 85 | 6 | 18 |
| Support vector machine (linear kernel) | 25 | 82 | 9 | 19 |
| Support vector machine (radial basis function) | 25 | 83 | 8 | 19 |
| Extreme Gradient Boosting | 25 | 76 | 15 | 19 |

Note: TP: Ture positive; TN: True negative; FP: False positive; FN: False negative;
